# Trends and variation in use of end-tidal carbon dioxide during in-hospital cardiac arrest: an observational cohort study

**DOI:** 10.3389/fmed.2026.1868798

**Published:** 2026-06-18

**Authors:** Luke Andrea, Joshua M Kimbrell, Shilpa Kolli, Michelle M Nassal, Ari Moskowitz

**Affiliations:** 1 Montefiore Medical Center, Bronx, NY, United States; 2 Newark Beth Israel Medical Center, Newark, NJ, United States; 3 Department of Emergency Medicine, The Ohio State University Wexner Medical Center, Columbus, OH, United States

**Keywords:** airway confirmation, American Heart Association guidelines, cardiopulmonary resuscitation monitoring, end-tidal carbon dioxide, in-hospital cardiac arrest

## Abstract

**Background:**

The American Heart Association recommends using end-tidal carbon dioxide during cardiac arrest for advanced airway confirmation and for monitoring and optimizing cardiopulmonary resuscitation; practice patterns of its use during in-hospital cardiac arrest are not well described.

**Methods:**

We used the prospectively collected get with the guidelines-resuscitation registry to characterize trends and hospital-level variation in end-tidal carbon dioxide use for airway confirmation in arrests with a newly placed airway, and to monitor cardiopulmonary resuscitation in arrests with a new or prior airway. Proportions were displayed by month from 2000 to 2023. Hospital use rates were displayed visually and calculated for contemporary practice (2019–2023). Proportions were reported using means or medians depending on the distribution.

**Results:**

Among 257,057 in-hospital cardiac arrests with a new airway, 92.6% were confirmed with end-tidal carbon dioxide in 2019–2023. Among the 554,693 in-hospital cardiac arrests with either a new airway or an airway already in place, 35.3% had end-tidal carbon dioxide applied for monitoring cardiopulmonary resuscitation in 2019–2023. There was little variation in hospital proportions for airway confirmation, with a median of 94.0%; however, a high degree of variation was observed across hospitals for monitoring cardiopulmonary resuscitation.

**Conclusion:**

End-tidal carbon dioxide application is widespread for confirming airway placement during in-hospital cardiac arrest, but gaps remain in adherence to guideline recommendations for monitoring cardiopulmonary resuscitation with end-tidal carbon dioxide.

## Introduction

**Figure 1:**
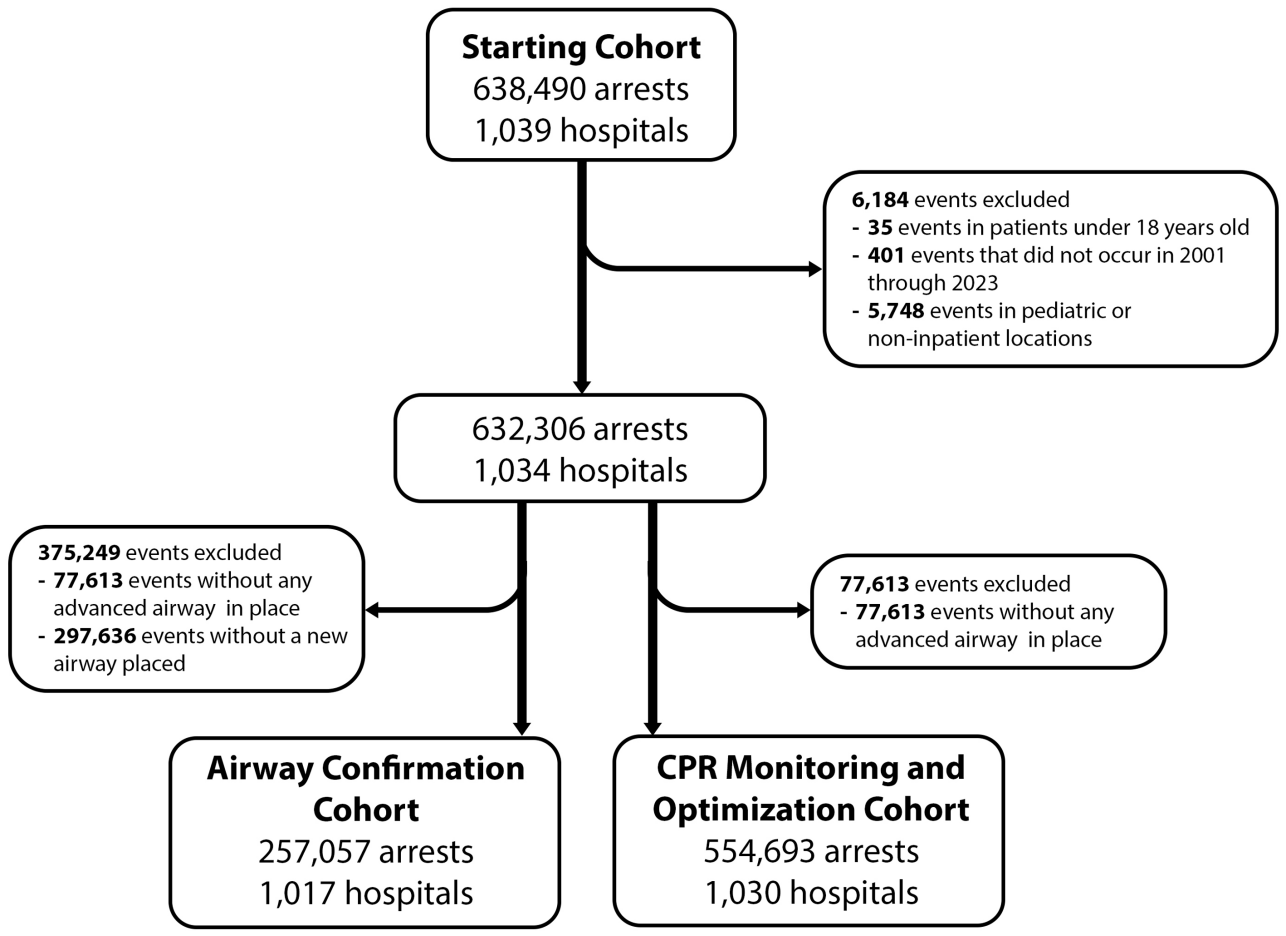
Cohort flow diagram.

The American Heart Association (AHA) recommends measuring end-tidal carbon dioxide (EtCO_2_, the concentration of carbon dioxide in exhaled air at the end of exhalation) for two separate indications during cardiac arrest. First, to confirm advanced airway placement, specifically recommending the use of waveform capnography due to its high sensitivity and specificity (1). Second, to optimize cardiopulmonary resuscitation (CPR) (1). EtCO_2_ reflects pulmonary blood flow, serving as a real-time, noninvasive surrogate for cardiac output and allowing monitoring of CPR quality. Abrupt, sustained increases in EtCO_2_ during CPR have been established as an indicator for detecting return of spontaneous circulation (ROSC). Persistently low EtCO_2_ (<10 mmHg) during CPR is consistently associated with unsuccessful resuscitation, and has been reported in the guidelines as a consideration when deciding on termination of resuscitation. These two distinct applications of EtCO_2_ during cardiac arrest, airway confirmation and CPR monitoring, have been recommended since 2005, with updates (Figure 1) in each guideline release (1–4). Nevertheless, practice patterns of EtCO_2_ use for in-hospital cardiac arrest (IHCA) are not well described.

## Methods

This brief research report is a registry-based, observational, descriptive study. We used the prospectively collected Guidelines® Resuscitation (GWTG®-R) registry; hospitals contributing to the database submit clinical information of consecutive patients with IHCA using an online case report form and Patient Management Tool™ (IQVIA, Parsippany, New Jersey). We included all adult (age ≥ 18 years) IHCA from 2000 to 2023. Arrests occurring in ambulatory and outpatient areas, rehab units, same-day surgical areas, or pediatric care areas were excluded.

We evaluated EtCO_2_ application in two separate utilization cohorts: (i) advanced airway confirmation, and (ii) CPR monitoring and optimization. The advanced airway confirmation cohort included only patients with a new airway placed during the arrest. New airway placement during IHCA was defined as a cardiac arrest for a patient who did not already have an advanced airway in place prior to the start of the arrest, and received an advanced airway after the arrest started. Data abstractors for GWTG®-R select an airway confirmation method when an advanced airway is placed. EtCO_2_ use for confirmation of airway placement was defined as the selection of colorimetry (a qualitative measurement that changes color in the presence of CO_2_), capnometry (a quantitative measurement of CO_2_ in exhaled gas), or waveform capnography (a quantitative measurement and display of the concentration of CO_2_ in exhaled gas over time) as the airway confirmation method within the GWTG®-R registry. The CPR monitoring and optimization cohort included all patients with an advanced airway, including those with an airway already in place. An airway already in place was defined as an advanced airway present for the patient prior to the start of the arrest. EtCO_2_ use for CPR monitoring was defined as the selection of any of the variables for CPR monitoring using EtCO_2_ within the GWTG®-R registry. Documenting the use of EtCO_2_ for CPR monitoring and optimization is optional in the GWTG®-R registry; we included any documented EtCO_2_ use for CPR monitoring in our analysis, and missing documentation was treated as non-use. The two utilization cohorts were not mutually exclusive.

The proportion of arrests where EtCO_2_ was applied was plotted monthly. We calculated proportions of EtCO_2_ application both overall and for contemporary practice (2019–2023). For proportions, 95% confidence intervals (95%CI) were calculated and reported. Means with standard deviations (SD) and medians with interquartile ranges (IQR) were reported based on the distribution of the data after visual inspection with histograms. Hospital-level proportions were calculated for 2019–2023, and plotted from lowest to highest; these years were selected to show contemporary hospital variation in EtCO_2_ use. All hospitals were included regardless of the number of IHCA entered into the database. For airway confirmation, in addition to overall EtCO_2_ application (colorimetry, capnometry, and waveform capnography), we separately calculated proportions of waveform capnography use.

We intended to characterize observed patterns in the cohort rather than evaluate prespecified hypotheses. Accordingly, all results were descriptive and presented using summary statistics; temporal patterns were evaluated descriptively through visual inspection of summary estimates over time. No comparisons or tests for trend were performed. All analyses were performed with Stata/SE version 19.0 (College Station, TX, StataCorp LP). Participating GWTG®-R hospitals are required to adhere to local regulatory and privacy guidelines. The Institutional Review Board (IRB) at the Albert Einstein College of Medicine deemed the use of the de-identified GWTG®-R as non-human subjects research (IRB#2025-16,709).

## Results

From 2000 to 2023, 638,490 IHCA occurred at 1,039 hospitals. After applying the inclusion and exclusion criteria, there were 632,306 total IHCA. From 1,034 hospitals (Figure 1).

**Figure 2:**
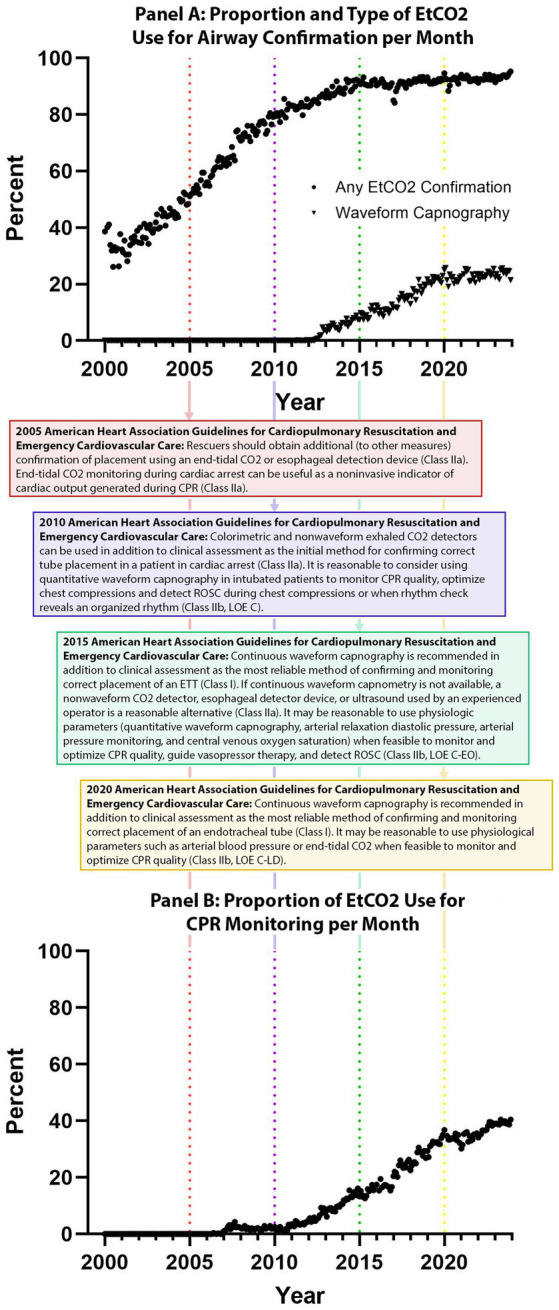
EtCO_2_ application during in-hospital cardiac arrest over time. EtCO_2_ use in each month for airway confirmation and CPR monitoring.

**Figure 3:**
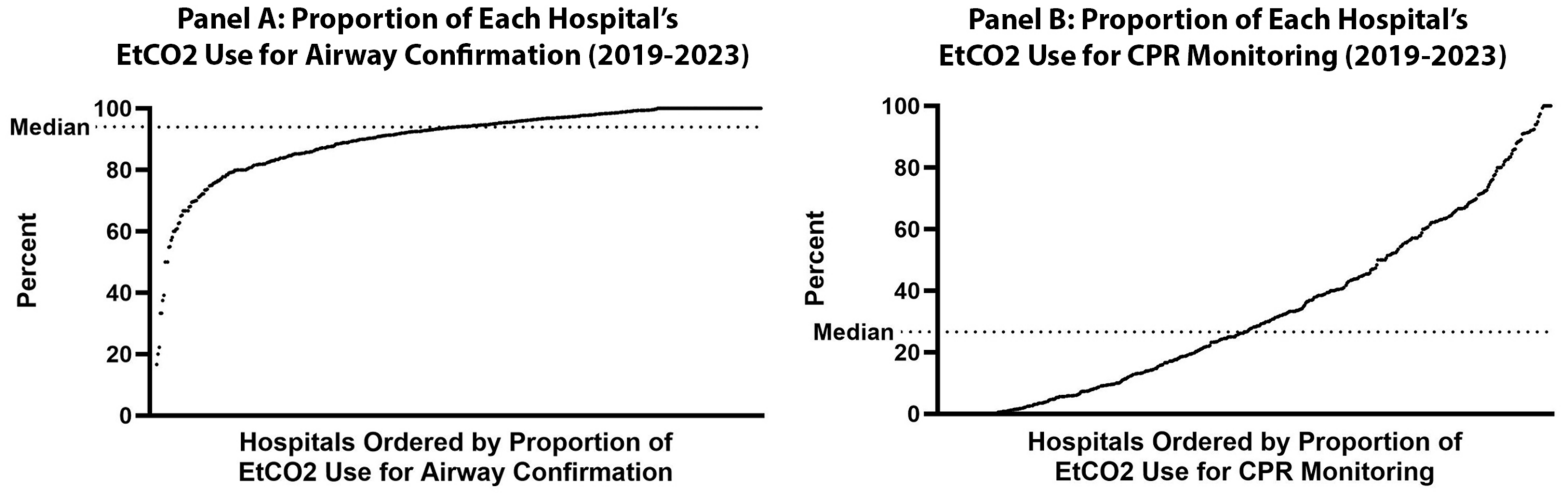
Hospital variation in EtCO_2_ application during in-hospital cardiac arrest. Variation in hospital application of EtCO_2_ in a contemporary cohort for airway confirmation (517 hospitals) and CPR monitoring (517 hospitals).

We identified 257,057 arrests where a new advanced airway was placed for our advanced airway confirmation cohort. Overall, 80.1% (205,917/257,057, 95%CI 80.0–80.3%) of new airways during IHCA had EtCO_2_ confirmation, with a mean proportion of 92.6% (73,844/79,741, 95%CI 92.4–92.8%) during the years of 2019–2023. Proportions of EtCO_2_ use for airway confirmation over time are displayed in Figure 2 Panel A. When evaluating EtCO_2_ airway confirmation by location of arrest, all inpatient locations had >90% use in 2019–2023 (Table 1). For the AHA-recommended method of advanced airway confirmation, waveform capnography, the mean proportion was 10.1% (25,978/257,057), rising to 22.7% (18,127/79,741) for 2019–2023. Capnometry was used to confirm 12.3% (31,522/257,057) of airways overall, and 27.8% (22,196/79,741) for 2019–2023. Colorimetry was used to confirm 68.9% (177,194/257,057) of airways overall, and 67.1% (53,497/79,741) for 2019–2023. The airway confirmation methods were not mutually exclusive in the dataset. Variation in hospital application of EtCO_2_ for airway confirmation from 2019 to 2023 (517 hospitals) is in Figure 3 Panel A; the median proportion was 94.0% (interquartile range 85.5–98.4%).

**Table 1:** EtCO_2_ use by arrest location.

Arrest location	EtCO_2_ not applied for airway confirmation *N* = 51,140	EtCO_2_ was applied for airway confirmation *N* = 205,917	EtCO_2_ not applied for airway confirmation (2019–2023)	EtCO_2_ was applied for airway confirmation (2019–2023)
Intensive care unit	8,039 (11.8%)	60,197 (88.2%)	1,563 (6.5%)	22,383 (93.5%)
Emergency department	6,433 (18.9%)	27,549 (81.1%)	1,279 (9.4%)	12,304 (90.6%)
Wards	22,577 (19.4%)	93,816 (80.6%)	2,360 (6.7%)	32,693 (93.3%)
Other	14,091 (36.7%)	24,355 (63.3%)	695 (9.7%)	6,464 (90.3%)

Arrest location	EtCO_2_ not applied for CPR monitoring *N* = 459,836	EtCO_2_ was applied for CPR monitoring *N* = 94,857	EtCO_2_ not applied for CPR monitoring (2019–2023) *N* = 114,579	EtCO_2_ was applied for CPR monitoring (2019–2023) *N* = 62,426
Intensive care unit	211,783 (78.8%)	56,922 (21.2%)	58,851 (61.4%)	37,000 (38.6%)
Emergency department	53,813 (81.7%)	12,089 (18.3%)	17,648 (66.7%)	8,816 (33.3%)
Wards	116,041 (88.1%)	15,666 (11.9%)	29,452 (74.0%)	10,337 (26.0%)
Other	78,199 (88.5%)	10,180 (11.5%)	8,628 (57.9%)	6,273 (41.1%)

Our CPR monitoring and optimization cohort included 554,693 IHCA cases with any advanced airway present, either already in place at the time of arrest or newly placed. Overall, the proportion of IHCA with EtCO_2_ application for CPR monitoring was 17.1% (94,857/554,693, 95%CI 17.0–17.2%), rising to a mean proportion of 35.3% (62,426/177,005, 95%CI 35.0–35.5%) for 2019–2023. Proportions of EtCO_2_ CPR monitoring over time are in Figure 2 Panel B. When evaluating EtCO_2_ CPR monitoring by location of arrest, the intensive care unit (37,000/95,851 [38.6%]) and emergency department (8,816/26,464 [33.3%]) both had higher proportions of use than the hospital wards (10,337/39,789 [26.0%]) in 2019–2023 (Table 1). Variation in hospital application of EtCO_2_ for CPR monitoring from 2019 to 2023 (517 hospitals) is shown in Figure 3 Panel B; the median proportion was 26.7% (interquartile range 8.0–53.5%).

## Discussion

We found that documented EtCO_2_ application for airway confirmation during IHCA is widespread in this large, U. S. cohort; however, adherence to the AHA-recommended method, waveform capnography, remains low. Documented application of EtCO_2_ for CPR monitoring, though increasing, remains limited and variable across hospitals.

Confirming advanced airway placement is an important component of managing cardiac arrest; esophageal intubation has been reported in over 5% of all first-pass tracheal intubation attempts (5). Prior reports have identified absent or inadequate EtCO_2_ confirmation as a common factor in unrecognized esophageal intubations (5, 6). However, several concerns have been raised regarding the use of EtCO_2_ to confirm tracheal tube placement, including the requirement of several detection breaths for confirmation which can increase the risk of aspiration if the tube is not properly placed, pre-existing gastric CO_2_ interfering with interpretation, and conditions that reduce CO_2_ production or delivery to the pulmonary circulation (including cardiac arrest) (7). The AHA guidelines specifically recommend waveform capnography to confirm endotracheal tube position during cardiac arrest, due to its high specificity during cardiac arrest (1, 8–10). Continuous waveform capnography offers advantages over other methods of EtCO_2_ airway confirmation due to its ability to provide real-time continuous visualization for detection of displacement or dislodgement during ongoing resuscitation efforts, rather than confirming placement at a single time point.

In our observational study of over 250,000 IHCA where a new airway was placed, overall proportions of airway confirmation using EtCO_2_ are high and have plateaued above 90%, with few hospitals documenting proportions below 80%. These findings reflect substantial success in implementation for this indication. The GWTG®-R registry has a public recognition program for participating hospitals, which partly depends on the proportion of documented airway confirmation during IHCA (11). Although this GWTG®-R program allows for airway confirmation methods other than EtCO_2_, incentives through this registry-based recognition may be a driver of the high documentation rates we identified. The AHA guidelines have recommended waveform capnography since 2015 as the preferred method for EtCO_2_ airway confirmation, but in our analysis, waveform capnography represents a minority among EtCO_2_ methods and has not increased in recent years, making it a potential area of future quality improvement initiatives.

Beyond airway confirmation, EtCO_2_ has clinical relevance for monitoring and optimizing CPR, though evidence supporting some of its various applications remains mixed. A propensity-matched analysis from the GWTG®-R registry found that monitoring CPR quality with EtCO_2_ was associated with higher odds of ROSC and survival to discharge, and AHA guidelines have consistently recommended EtCO_2_ use for this indication (12). Trends in EtCO_2_ also provide some of the earliest noninvasive signals of ROSC, and have been suggested as a marker for low probability of surviving an arrest (13, 14). Conversely, a recent systematic review and meta-analysis by Lee et al. supports a more nuanced picture, with limited prognostic accuracy from EtCO_2_ levels (13). When considering termination of resuscitation (ToR), the AHA does not recommend using EtCO_2_ values in isolation, however there is some evidence, such as that by Kudu et al., that suggests integrating EtCO_2_ values into validated ToR rules for out-of-hospital arrests may increase the specificity (1, 15). Unfortunately, well-validated ToR rules do not exist for IHCA (16), which is the focus of this study on EtCO_2_ use. In our analysis of 554,693 IHCA where an airway was in place, we found that EtCO_2_ application for CPR monitoring is generally low, and highly variable across hospitals, with use appearing to be increasing in recent years. Documentation of this parameter in the GWTG®-R registry is optional; therefore, the proportions we present may underrepresent true rates. Further, EtCO_2_ is only one measure of CPR quality, and this analysis does not capture all CPR monitoring methods.

This description of EtCO_2_ use during IHCA has several limitations. First, the observed patterns of EtCO_2_ application are limited to hospitals participating in the GWTG®-R registry; hospitals outside of the registry are not subject to GWTG®-R incentives, and their practice patterns remain unknown. Second, participating hospitals may vary in how they record CPR quality data, contributing to observed differences. If there is documentation bias, it likely underestimates true use, especially for CPR monitoring where there is no incentive in place. Third, proportions presented here represent only documented cases and may not fully capture actual clinical application if EtCO_2_ was used but not documented, resulting in potential misclassification bias. Fourth, we included all hospitals, regardless of the number of cardiac arrests that they enrolled; this decision was made because adherence to guideline-recommended EtCO_2_ application is expected irrespective of institutional arrest volume, and excluding low-volume centers could bias estimates of real-world practice patterns. The decision to include low-volume centers, however, may also exaggerate apparent variation, a potential limitation. Fifth, we do not have information on the reasons that EtCO_2_ was not applied or not documented during cardiac arrest. Identifying potential barriers to EtCO_2_ application such as device application or clinician behavior, may be beneficial for future implementation efforts. Finally, we do not present information on patient outcomes or hospital characteristics, information that may be beneficial for future investigations aiming to improve adherence to EtCO_2_ guidelines during IHCA.

Our findings highlight gaps in adherence to two separate AHA guidelines for EtCO_2_ use in IHCA, and help contextualize trends in EtCO_2_ application and current practice during IHCA. The trends and variation we identified, such as differences in adherence in different hospital locations, may be helpful for future implementation and comparative effectiveness research.

## Data Availability

Publicly available datasets were analyzed in this study. This data can be found at: get with the guidelines-resuscitation.
